# Genome sequence of *Vibrio* sp. strain 10N, an ulvan-degrading bacterium isolated from coastal seawater collected at Ise Bay, Japan

**DOI:** 10.1128/mra.00972-23

**Published:** 2024-01-11

**Authors:** Reiji Tanaka, Hideo Miyake, Toshiyuki Shibata

**Affiliations:** 1 Graduate School of Bioresource, Mie University, Tsu, Japan; 2 Seaweed Biorefinery Research Center, Mie University, Tsu, Japan; University of Southern California, Los Angeles, California, USA

**Keywords:** ulvan, green alga, *Vibrio*, seawater

## Abstract

Genome sequence of an ulvan-degrading bacterium, *Vibrio* sp. strain 10N, is presented. The genome is 5,358,550 bp with a G + C content of 46.5%. A total of 4,712 coding sequences, including two novel ulvan lyase genes encoding a BNR4 and a glycoside hydrolase (GH88) motif, are known to be involved in the degradation of green algae.

## ANNOUNCEMENT

Green algae-degrading bacteria have recently been considered to be important targets for biorefinery because they are a key source for the assimilation of ulvan, a major component of polysaccharide in green algae that is currently used as a bioresource for the production of bioethanol (1). Ulvan-degrading bacteria are also an important source for evolutionary studies to understand the as-of-yet unclear mechanisms behind ulvan assimilation (2, 3).

**Fig 1:**
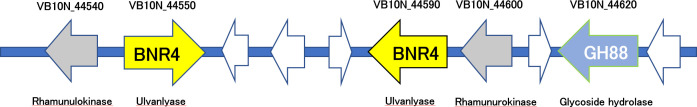
Chromosome 1 of a putative ulvan-degrading-related gene of *Vibrio* sp. strain 10N. Yellow arrows indicate BNR4 motif protein (VB10N_44550, 45590). Light blue arrow indicates glycoside hydrolase family GH88 (VB10N_44620). White arrows indicate hypothetical protein, and gray arrows indicate genes that are not relevant to ulvan degradation.

Here, we report the draft genome sequence of a novel ulvan-degrading bacterium, *Vibrio* sp. strain 10N. This bacterium was isolated in September 2018 from Ise Bay (34°44*'*32*"*N; 136°31*'*48*"*E) in Mie, Japan, and the strain was sequenced and described here (4, 5). Strain 10N stored at −80°C was inoculated into Marine broth 2216 (BD Difco, MD, USA) and shaken at 25°C, 100 rpm for 24 h. The genome of strain 10N was extracted according to the manufacturer’s instructions using DNA purification system (Promega, MD, USA) and used for both with the IIlumina Novaseq set and GridION platform. Libraries for Novaseq were prepared as described in the manual using fragmented DNA (500 ng) and the KAPA HyperPrep Kit (Roche, Swiss land). Adapters from the FastGene Adapter Kit (Nippon Genetics, Japan) were used, and the library was amplified by six cycles of PCR. We removed each base with a quality value of less than 20 from the reads sampled using Sickle (Ver1.33) (https://github.com/najoshi/sickle) and discarded the reads and their paired reads that had a quality value of less than 127 bases. The library for GridION was prepared using the Ligation Sequence Kit (SQK-LSK 109) with DNA (1,500 ng) as input, but any size selection was not done prior to the sequencing. R.9.4.1 flow cell was used for the analysis. Base calling for the raw reads was performed using Guppy (Ver3.3.3) by fast calling without high-accuracy model. Porechop (Ver0.2.3) (https://github.com/rrwick/Porechop), Filtlong (Ver0.2.0) (https://github.com/rrwick/Filtlong), and Canu (ver 1.8) (6) were used for trimming, filtering, and correction, respectively. Default parameters were used for all software unless otherwise specified. In total, 5,369,718 sequence reads were obtained from the Novaseq platform, and 359,396 sequence reads with an average length of 10,690 bp were obtained from the GridION platform. Raw read N50 was 28,475 bp. Hybrid genome assembly using data from Novaseq and GridION was performed with the Unicycler system (Ver0.4.7) (7). The genome consists of two chromosomes and two plasmids, all of which are circular and have a total length of 5,358,550 bp, the G + C content was 46.5%, and the genome sequence contained 4,712 coding sequences; in addition, 112 tRNA genes for amino acids and 34 rRNA genes (short subunit, 11; long subunit, 11; 5S, 12) were identified through DFAST (ver1.2.0) (8). NCBI-blastn of its 16S rRNA gene showed 98.5% similarity to *Vibrio maritimus* strain R-40493 (GU-929925.1). Gene annotation and an overview of the annotated genome were viewed using DFAST (ver1.2.0) (8). We determined two ulvan lyases which have high similarity to sequences of BNR4-like protein and a glycoside hydrolase family GH88 (Fig. 1).

## Data Availability

The whole-genome sequence has been deposited in the DDBJ/EMBL/GenBank under the accession number BTPM01000000. The version described in this paper is version 1.00. The WGS data including annotation have been deposited in the DDBJ/EMBL/GenBank under the accession numbers BTPM01000001, BTPM01000002, BTPM01000003, and BTPM01000004. The BioProject number is PRJDB16227. The BioSample number is SAMD00631771 (*Vibrio* sp. 10N). Sequence Read Archive numbers are DRR493438 and DRR493439.
